# Social Support Modulates Neural Responses to Unfairness in the Ultimatum Game

**DOI:** 10.3389/fpsyg.2018.00182

**Published:** 2018-02-20

**Authors:** Chunli Wei, Li Zheng, Liping Che, Xuemei Cheng, Lin Li, Xiuyan Guo

**Affiliations:** ^1^School of Psychology and Cognitive Science, East China Normal University, Shanghai, China; ^2^Shanghai Key Laboratory of Magnetic Resonance, East China Normal University, Shanghai, China; ^3^Shanghai Key Laboratory of Brain Functional Genomics, Ministry of Education, East China Normal University, Shanghai, China; ^4^National Demonstration Center for Experimental Psychology Education, East China Normal University, Shanghai, China; ^5^Business School, University of Shanghai for Science and Technology, Shanghai, China; ^6^Department of Mechanical and Electrical Engineering, Beijing Polytechnic College, Beijing, China

**Keywords:** unfairness, ultimatum game (UG), social support, decision-making, fMRI

## Abstract

The current functional MRI study aimed to investigate how responders’ fairness considerations and related decision-making processes were affected by social support in the ultimatum game (UG). During scanning, responders either played the standard UG with proposers (control condition) or played the modified UG in which three unknown observers showed social support for responders by acknowledging proposers’ norm violation. Results revealed that participants reported higher unfairness feelings and rejection rates of unfair offers in the social support condition relative to the control condition. At the neural level, compared to the control condition, perception of social support from others induced greater activations of anterior cingulate gyrus and right anterior insula when receiving unfair (vs. fair) offers. The medial prefrontal cortex and right anterior insula were more active when the unfair offers were rejected (vs. accepted) in the social support condition than the control condition. These results highlighted the modulation effect of social support on responders’ fairness considerations and related decision-making processes.

## Introduction

Fairness-related decision-making has attracted much attention in the past decades and been widely studied by employing the Ultimatum Game (UG) (Güth et al., 1982; Camerer and Thaler, 1995; Sanfey et al., 2003; Civai et al., 2012; Guo et al., 2014; Hu et al., 2016). This game was developed by Güth et al. (1982), in which two players have to divide a sum of money according to the simple rule. One player proposes how to split and the other player responds (i.e., the proposer and the responder). The responder can either accept or reject the proposal. If the proposal is accepted, both players get the amount specified in the proposal. If the proposal is rejected, none of them receives any money. It has been documented in previous studies that responders accepted all fair offers, but often rejected extremely unfair offers (Güth et al., 1982; Camerer and Thaler, 1995). This results appeared in contradiction to the standard economic models, which idealized individuals as completely rational cognitive agents aiming to maximize their own payoff and assumed that the responder should accept any offer as long as it is larger than zero. The reason why people make such irrational decisions has been attributed to the negative emotion caused by perception of unfairness, people’s preference for fairness and tendency to maintain fairness norms (Bolton and Rami, 1995; Nowak et al., 2000; Sanfey et al., 2003; Yamagishi et al., 2009).

Over the past few years, a large body of neuroimaging studies have investigated the neural basis underlying the fairness-related decision-making processes and identified the engagement of several brain regions, including anterior insula (AI), anterior cingulate cortex (ACC), amygdala and prefrontal cortex (Sanfey et al., 2003; Haruno and Frith, 2010; Güroğlu et al., 2011; Civai et al., 2012; Corradi-Dell’Acqua et al., 2013). It has been proved that the activations of AI and ACC observed during receiving and rejecting unfair offers are associated with detecting and responding to fairness norm violations (Sanfey et al., 2003; Chang and Sanfey, 2013; Corradi-Dell’Acqua et al., 2013; Xiang et al., 2013; Guo et al., 2014). Amygdala has been found playing a key role in emotional processing (Scott et al., 1997; Rauch et al., 2003; Feinstein et al., 2011), and its activation in UG was suggested to be related to inequity aversion (Haruno and Frith, 2010). As for the prefrontal cortex, previous studies have observed the activation of the dorsal lateral prefrontal cortex (DLPFC) and medial prefrontal cortex (mPFC) during fairness-related decision processes (Sanfey et al., 2003; Baumgartner et al., 2011; Civai et al., 2012; Corradi-Dell’Acqua et al., 2013; Cheng et al., 2015). The activation of DLPFC was interpreted to be engaged in the integration of information and the selection of context-appropriate decisions to unfairness (Buckholtz et al., 2008; Buckholtz and Marois, 2012; Cheng et al., 2015). The mPFC has been thought to be involved in monitoring one’s behavioral responses in social decision-making (Civai et al., 2012, 2015; Corradi-Dell’Acqua et al., 2013).

As a kind of complex social interactions, responders’ fairness-related decision-making processes were not only determined by the proposal he or she received, but also influenced by various social contexts, such as the social distance between proposers and responders (Wu et al., 2011), the framing of distribution (Zhou and Wu, 2011; Guo et al., 2013), self-contribution to the income (Guo et al., 2014), proposers’ economic status (Zheng Y. et al., 2017) and so on. The present study will investigate whether one of these contextual factors, social support, modulates peoples’ fairness-related decision making, behaviorally and neurally. Social support refers to the mental and material resources which people obtained from the social network, including sympathy, caring, actions, advice, information (Cobb, 1976; Thoits, 1986). In UG, the responders were at relative disadvantage positions, hence resulted in negative emotional feelings in them (Sanfey et al., 2003; Wout et al., 2006). Social support has been identified as having a critical impact on people’s psychological state and behaviors when they are under negative emotional states (Cohen and Wills, 1985; Sarason et al., 1997). It has been found that social support can help people cope with stress situations, cease smoking and alcohol consumption (Cohen and Wills, 1985; Steptoe et al., 1996; Burns et al., 2014), and has beneficial effects on one’s well-being, physical and psychological health (Turner, 1981; Uchino et al., 2016). However, little researchers have discussed its impact on fairness-related decision-making behaviors, less for the underlying neural mechanisms.

To explore the modulation effect of social support on responders’ fairness-related decision-making process and the underlying neural mechanisms, we designed the current functional magnetic resonance imaging (fMRI) study. During the experiment, the participants carried out both the standard version of UG (control condition) and a modified version of UG (social support condition) as responders in the scanner. According to the typical laboratory manipulation of social support, which employ support providers who deliver emotional support to participants by verbal comments, such as expressions of blaming the norm violators (Cutrona and Russell, 1990; Thorsteinsson and James, 1999; Cohen et al., 2000), in the present study, there are support providers delivering social support for the participants by acknowledging the proposer’s fairness norm violations and having themselves at the participants’ back.

Based on the emerging evidence, there might be two different hypothesizes on how social support would modulate responders’ fairness-related decision-making processes. On the one hand, researchers have argued that responders’ rejection is driven by the negative emotions evoked by unfair treatment (Sanfey et al., 2003; Wout et al., 2006; Yamagishi et al., 2009). Social support has been demonstrated as being able to alleviate people’s negative emotions effectively (Cohen and Wills, 1985; Sarason et al., 1997), thus the responder’s unfairness-related negative emotional feelings might decrease when receiving others’ social support. Decreased rejection rates and amygdala activation might also be observed. We called it as the “negative emotion buffer” hypothesis. On the other hand, some prior studies have pointed out that people were easily infected by other’s attitudes (Prislin and Wood, 2005; Huang et al., 2014). The social support supplied to the responder by verbal comments implied that the support providers confirmed the proposer’s violations to fairness norm. In this case, the responder might be influenced by attitudes of supporters and be more sensitive to the violations of fairness norms, showing increased unfairness-related negative emotional feelings and rejection rates to unfair offers under the social support condition, accompanied with increased activations of AI and ACC. This was called as the “norm violation confirmation” hypothesis.

## Materials and Methods

### Participants

Twenty-eight right-handed volunteers [15 females, mean age = 22.46 (years), *SD* = 2.62 (years)] from the university community participated in this experiment. None of the participants had an abnormal neurological history. All of them had normal or corrected-to-normal vision. Three participants were excluded from further statistical analyses. One participant was excluded due to a technical problem during scanning and the other two had severe head movements (>3 mm or 3°) (Cheng et al., 2015; Nebel et al., 2016). Written informed consent was acquired from all participants before scanning. This study was approved by the Ethics Committee on Human Experiments of East China Normal University.

### Procedure

Before scanning, participants were told the rules of the game and that they would receive proposals about how to divide 50 RMB from 72 different proposers whose proposals were collected before the experiment. In half of the trials, participants acted as the responder and played the standard UG with the proposer. For the standard UG, the proposer gave her/his division schema about a sum of money and the responder decided to accept or reject it (control condition). While in the other half trials, participants were supported by three unknown observers when playing the UG with the proposer (social support condition). In the social support condition, participants were told that the observers acknowledged the proposer’s fairness norm violations and had themselves at their back. As for the payment, participants were informed that several trials would be randomly selected and that both they and the proposers would be paid according to their decisions. Finally, participants would be paid with the amount of money obtained from a random selection of 5% trials in the game plus a 50RMB (approximately equal to 32 dollars) bonus. In fact, the proposals were manipulated by the experimenter and there were no real proposers or supporters. One hundred and eighty female or male neutral face pictures were randomly selected from the Chinese Affective Face Picture System (Gong et al., 2011) were used as the proposers and the supporters in different contexts.

**FIGURE 1:**
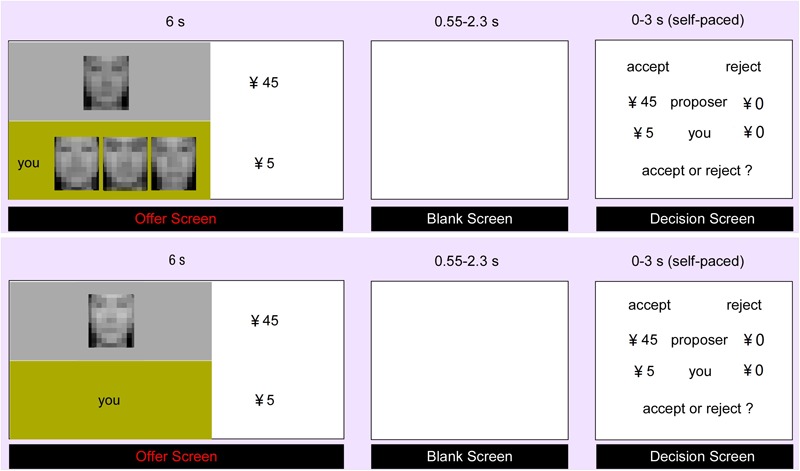
Experimental Procedure. The participant firstly received the offer from the proposer (social support context in the upper part and control context in the lower part). After a jittered blank lasting for 0.55–2.3 s, the participant was asked to decide to accept or reject the offer within 3 s. During the experiment, all the face pictures presented to the participant were clear without mosaic.

Then, the participants completed 72 trials in the scanner. There were 36 trials in each context, including 12 fair trials (25:25) and 24 unfair trials. The unfair trials contained four types of proposals, i.e., 30:20, 35:15, 40:10 and 45:5, with each type having 6 trials. All the trials were presented randomly and functional images were acquired simultaneously. Each trial began with the presentation of the proposer’s offer, which lasted for 6 s. At the same time, context information about whether participants had supporters or not would also be presented. Then a blank screen jittered from 0.55 ∼ 2.3 s was presented. After that, participants were required to decide (accept or reject) within 3 s. Each trial was jittered with inter-stimulus intervals (approximately 3 ∼ 8 s), during which a black fixation cross was presented (**Figure 1**). After scanning, the same stimuli as inside the scanner were presented again. Participants were asked to rate the extent of unfairness-related negative emotional feelings they felt for each offer (i.e., unfairness ratings) in a 9-point Likert-type scale (1 indicated extremely unfair and 9 indicated extremely fair).

### fMRI Image Acquisition and Data Analyze

The scanning was carried out on a 3T Siemens scanner at the Shanghai Key Laboratory of Magnetic Resonance of East China Normal University. Anatomical images were acquired using a T1-weighted, multiplanar reconstruction sequence (MPR) (TR = 1900 ms, TE = 3.42 ms, 192 slices, slice thickness = 1 mm, FOV = 256 mm, matrix size = 256 ^∗^ 256). After that, functional images were acquired using a gradient echo echo-planar imaging (EPI) sequence (TR = 2200 ms, TE = 30 ms, FOV = 220 mm, matrix size = 64 ^∗^ 64, 35 slices, slice thickness = 3 mm, gap = 0.3 mm).

Participants’ data were analyzed using the SPM8 software package (Wellcome Department of Imaging Neuroscience, London, United Kingdom). During data preprocessing, the first five volumes were discarded to allow for T1 equilibration effects. Then, the functional images were corrected for the delay in slice acquisition and were realigned to the first image to correct for interscan head movements. The individual structural image was co-registered to the mean EPI image generated after realignment. The co-registered structural image was then segmented into gray matter (GM), white matter (WM) and cerebrospinal fluid (CSF) using a unified segmentation algorithm (Ashburner and Friston, 2005). The functional images after slice timing correction and realignment procedures were spatially normalized to the Montreal Neurological Institute (MNI) space (resampled at 2 mm × 2 mm × 2 mm voxels) using the normalization parameters estimated during unified segmentation and then spatially smoothed with a Gaussian kernel of 8 mm full-width half-maximum (FWHM).

First-level analyses were then performed across the whole brain for each subject using two general linear models (GLM) implemented in SPM8. The fairness-related model was built to explore the impact of social pressure on unfairness-related neural responses, consisting of four types of events (Fair*ss*: fair offers in the social support condition, Unfair*ss*, unfair offers in the social support context; Fair*cc*: fair offers in the control condition, Unfair*cc*, unfair offers in the control condition). Events were convolved with a canonical hemodynamic response function (HRF). All the encoding trials were time-locked to the onset of the offers with null duration. Decision phase and trials with no response were also added into the model as additional covariates of no interest. Moreover, six realignment parameters and one overall mean during the whole phase were included in the design matrix as well. To filter the low-frequency noise, a cutoff of 128 s was applied. Contrast images for each type of event (Fair*ss*, Unfair*ss*, Fair*cc*, Unfair*cc*) were computed for each participant at the first-level analysis. At the second group level, these four first-level individual contrast images were fed into a 2 (Context: social support condition vs. control condition) × 2(Unfairness: Unfair vs. Fair) factorial design using a random-effects model (flexible factorial ANOVA in SPM8). The main effect of unfairness was defined using the (Unfair – Fair) and the reverse contrasts. The interaction between unfairness and social context was defined by the (Unfair*ss* – Fair*ss*) – (Unfair*cc* – Faircc) and the reverse contrasts. A cluster-level threshold of *p* < 0.05 (family wise error corrected) and a voxel-level threshold of *p* < 0.001 (uncorrected) were used to define activations.

To explore how the neural correlates underlying people’s response to unfairness (rejection/acceptance) were modulated by social support, we built a response-related model in which unfair offers were further divided according to participants’ responses (UAss, accepted unfair offers in the social support condition, UR*ss*, rejected unfair offers in the social support condition; UA*cc*, accepted unfair offers in the control condition, UR*cc*, rejected unfair offers in the control condition). The rest of the analyses were carried out in the same way as those in the first model. Contrast images for four types of event (UA*ss*, UR*ss*, UA*cc*, UR*cc*) were computed for each participant at the first-level analysis and then fed into a 2 (Context: social support vs. control) × 2 (Response: UA vs. UR) flexible factorial using a random-effects model (flexible factorial ANOVA in SPM8). The main effect of response to unfairness was defined using the (UR – UA) and the reverse contrasts. The interaction between response and social context was defined by the (UR*ss* – UA*ss*) – (UR*cc* – UA*cc*) and the reverse contrasts. A cluster-level threshold of *p* < 0.05 (family wise error corrected) and a voxel-level threshold of *p* < 0.001 (uncorrected) were used to define activations.

In addition, parametric analyses, an efficient statistical procedure to reveal voxels that shows a particular pattern of activation throughout several conditions (Büchel et al., 1998), was conducted at the first-level to assess how brain activities were modulated by unfairness. Specifically, unfairness ratings were used as the parametric regressor separately for two social contexts. The resulting subject-specific estimates of the parametric regressor at each voxel were then entered into a second-level one sample t-tests. A cluster-level threshold of *p* < 0.05 (family wise error corrected) and a voxel-level threshold of *p* < 0.001 (uncorrected) were used to define activations.

## Results

### Behavior Results

The behavioral results were shown in **Table 1**. For rejection rates, participants accepted all the fair offers in both contexts. However, paired *t*-tests revealed higher rejection rates for unfair offers in the social support condition than those in the control condition [*t*(27) = 8.25, *p* < 0.001]. For unfairness ratings, a 2 (Fairness: Unfair vs. Fair) × 2 (Social context: social support vs. control) repeated-measure ANOVA revealed a significant main effects of fairness [*F*(1,27) = 1848.50, *p* < 0.001, $\eta_{p}^{2}$ = 0.99] and social context [*F*(1,27) = 45.52, *p* < 0.001, $\eta_{p}^{2}$ = 0.63], also a significant interaction [*F*(1,27) = 29.54, *p* < 0.001, $\eta_{p}^{2}$ = 0.52]. *Post hoc* analyses showed that unfairness ratings for unfair offers in the social support condition were lower relative to those in the control condition [*t*(27) = 7.97, *p* < 0.001], indicating participants’ stronger unfairness feelings in the social support condition. The current results of rejection rates and unfairness ratings were contrary to the “negative emotion buffer” hypothesis, while in line with the “norm violation confirmation” hypothesis.

**Table 1:** Mean (*SD*) for rejection rates (%) and unfairness ratings.

	Social support		Control	
	Fair	Unfair	Fair	Unfair
Rejection rates	0.00 (*0.00*)	77.00 (*0.08*)	0.00 (*0.00*)	62.00 (*0.10*)
Unfairness ratings	8.95 (*0.18*)	2.96 (*0.64*)	8.93 (*0.26*)	3.51 (*0.75*)

### fMRI Results

#### Main Effects

The main effect of Unfairness was tested by the (Unfair – Fair) and the reverse contrasts. Results showed stronger activations in right dACC, bilateral AI, left DLPFC, left supplementary motor area and left middle temporal gyrus during unfair compared to fair trials. No suprathreshold activation was detected in the reverse contrast. When contrasting trials in the social support condition with trials in the control condition, significant activations in right calcarine gyrus, right inferior frontal and left precentral were revealed. The reverse contrast revealed significant activations in left superior temporal gyrus, right superior temporal gyrus and left Cuneus. The main effect of response computed by the (UR – UA) contrast revealed significant activations in bilateral putamen, bilateral supramarginal gyrus and right supplementary motor area. The reverse contrast revealed no suprathreshod activations (**Table 2**).

**Table 2:** Brain activities showing unfairness, context and response main effects.

Region	Side	Peak activation			*t*-value	Voxels
		*X*	*Y*	*Z*		
**(Unfair – Fair)**
Supplementary motor area	L	-4	18	50	11.28	49083
*Dorsal anterior cingulate cortex*	R	8	26	36	9.88	
*Insula lobe*	L	-32	22	4	9.05	
*Insula lobe*	R	32	24	2	8.08	
*Dorsolateral prefrontal cortex*	L	-46	38	30	7.81	
Middle temporal gyrus	L	-46	2	-26	5.88	273
**(Fair – Unfair)**
No regions						
**(Social support – Control)**						
Calcarine gyrus	R	16	-90	2	15.8	22274
Inferior frontal gyrus	R	42	10	34	8.58	3084
Precentral gyrus	L	-36	0	50	6.11	920
**(Control – Social support)**						
Superior temporal gyrus	L	-54	-32	12	5.06	802
Superior temporal gyrus	R	66	-10	4	4.51	736
Cuneus	L	-4	-90	24	5.36	316
**(UR – UA)**						
Putamen	L	-22	10	0	5.84	1082
Putamen	R	28	8	12	6.25	745
Supramarginal gyrus	R	60	-26	24	4.64	395
Supplementary motor area	R	14	-10	68	4.31	357
Supramarginal gyrus	L	-52	-36	28	4.48	268
**(UA – UR)**						
No regions						

*Coordinates (mm) are in MNI space. L, left hemisphere; R, right hemisphere. Cluster–level, *p* < 0.05, family wise error corrected, voxel-level, *p* < 0.001, uncorrected.*

#### Unfairness–Related Effects: Context × Unfairness Interaction

**FIGURE 2:**
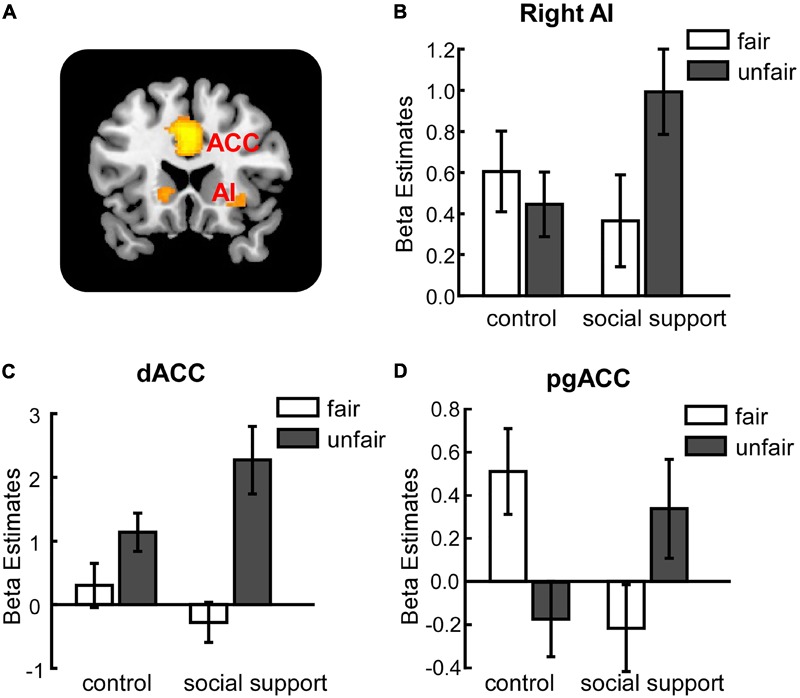
Unfairness-related activations in right AI **(B)**, dACC **(C)** and pgACC **(D)** were modulated by social support. **(A)** The activation map. AI, anterior insula. dACC, dorsal anterior cingulate cortex. pgACC, pregenual anterior cingulate cortex. Error bars indicated 95% confidence intervals. Cluster level, *p* < 0.05, family wise error corrected; voxel level, *p* < 0.001, uncorrected.

The interaction between context and unfairness computed by the (Unfair*ss* – Fair*ss*) – (Unfair*cc* – Fair*cc*) contrast showed stronger activations in right AI, dACC and pgACC. No significant activations were revealed in the reverse contrast. The activation of amygdala wasn’t observed in these two contrasts even at the uncorrected threshold (**Table 3**). Beta values in different conditions were extracted from all the significant voxels in the 6 mm-radius spherical regions centered on AI (MNI 26 24 -10), dACC (MNI -4 32 28) and pgACC (MNI 12 40 0) (beta values were extracted in the same way throughout the paper). As shown in the **Figure 2**, the activations of AI and dACC were stronger in the social support condition than those in the control condition for unfair offers [AI (**Figure 2B**): *F*(1,24) = 7.86, *p* < 0.05, $\eta_{p}^{2}$ = 0.25; dACC (**Figure 2C**): *F*(1,24) = 23.02, *p* < 0.001, $\eta_{p}^{2}$ = 0.49], which was consistent with the “norm violation confirmation” hypothesis. The pgACC was more active for fair offers compared with unfair offers in the control condition [*F*(1,24) = 10.81, *p* < 0.05, $\eta_{p}^{2}$ = 0.31], while this pattern was almost reversed in the social support condition [*F*(1,24) = 3.46, *p* > 0.05, $\eta_{p}^{2}$ = 0.13] (**Figure 2D**).

**Table 3:** Brain activities showing context × unfairness interaction and context × response interaction.

Region	Side	Peak activation			*t*-value	Voxels
		*X*	*Y*	*Z*		
**(Unfair*ss*– Fair*ss*) – (Unfair*cc*– Fair*cc*)**
Pregenual anterior cingulate cortex	R	12	40	0	6.33	7631
*Dorsal anterior cingulate cortex*	L	-4	32	28	6.31	
Thalamus	R	4	-12	8	5.46	2460
*Anterior insula*	R	26	24	-10	4.31	
Rolandic operculum	R	56	-6	12	5.04	1397
Heschls gyrus	L	-38	-20	4	5.24	1047
Temporal pole	L	-54	12	-2	4.41	226
**(Unfair*cc* – Fair*cc*) –(Unfair*ss*–Fair*ss*)**
No regions						
**(URs*s* – UAs*s*) – (UR*cc* – UA*cc*)**
Inferior occipital gyrus	R	40	-72	-6	7.22	9966
Supplementary motor area	L	-8	0	62	6.54	4690
Medial prefrontal cortex	R	12	58	6	5.36	735
Precentral gyrus	R	30	-8	48	4.47	603
Inferior frontal gyrus	R	32	32	-8	4.91	302
*Anterior insula*	R	34	28	6	3.67	
**(UR*cc* – UA*cc*) – (URs*s* – UAs*s*)**
No regions						

*Coordinates (mm) are in MNI space. L, left hemisphere; R, right hemisphere. Cluster–level, *p* < 0.05, family wise error corrected, voxel-level, *p* < 0.001, uncorrected.*

#### Response–Related Effects: Context × Response Interaction

**FIGURE 3:**
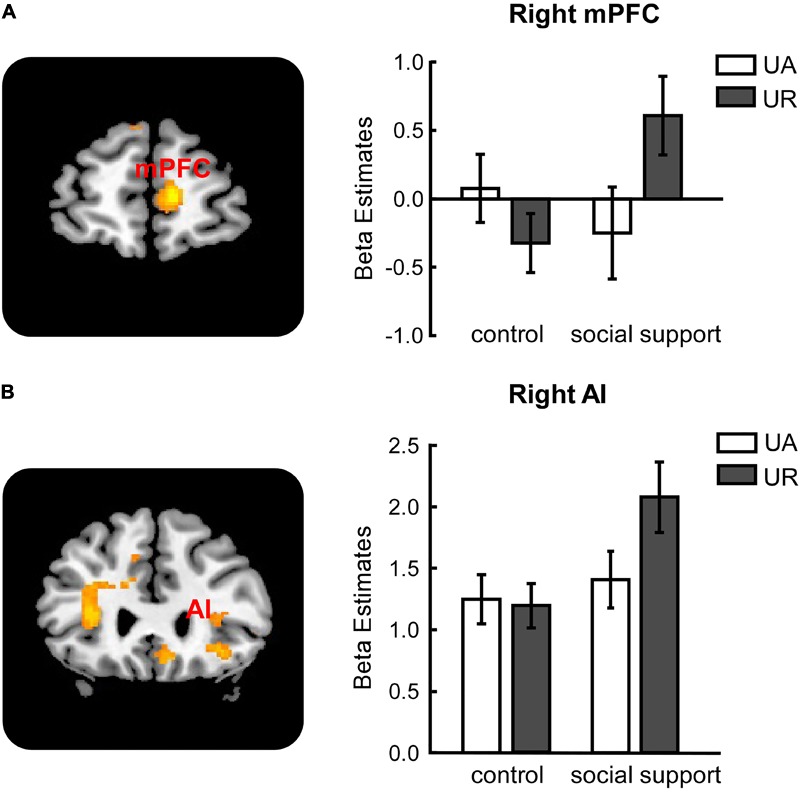
Response-related activations in right mPFC **(A)**, right AI **(B)** were modulated by social support. mPFC, medial prefrontal cortex. AI, anterior insula. UA, accepted unfair offers. UR, rejected unfair offers. Error bars indicated 95% confidence intervals. Cluster level, *p* < 0.05, family wise error corrected; voxel level, *p* < 0.001, uncorrected.

Significant activations in right mPFC and right AI were observed in the (URs*s* – UAs*s*) –(UR*cc* – UA*cc*) contrast (**Table 3**). The reverse contrast revealed no significant activation. Further analyses on beta estimates revealed that right mPFC and right AI were more active during rejecting relative to accepting unfair offers in the social support condition [right mPFC (**Figure 3A**), *F*(1,24) = 8.53, *p* < 0.05, $\eta_{p}^{2}$ = 0.26; right AI (**Figure 3B**), *F*(1,24) = 9.60, *p* < 0.05, $\eta_{p}^{2}$ = 0.29], but not in the control condition (*ps* > 0.05). Actually, amygdala also showed stronger activations when unfair offers were rejected in the social support condition compared with the control condition, though the voxel size (*k* = 107) failed to survive the current corrected criterion.

### Parametric Analyses on Unfairness Ratings

Parametric analyses on unfairness ratings revealed that left AI, right dACC (MNI 10 34 26) and left DLPFC (MNI -38 58 16) activations increased with the decrease of unfairness ratings in the social support condition and left AI (MNI -30 18 12) activation increased with the decreasing level of unfairness ratings in the control condition (**Table 4**). No suprathreshold activations were revealed with the increase of unfairness ratings.

**Table 4:** Regions showing increased activations with the decrease of unfairness ratings in two conditions.

Region	Side	Peak activation			*t*-value	Voxels
		*X*	*Y*	*Z*		
**Social support condition**						
Anterior insula	L	-42	14	8	6.05	5446
Supplementary motor area	L	-6	20	48	5.59	2332
*Anterior cingulate cortex*	R	10	34	26	4.53	
Superior parietal lobe	R	16	-62	60	4.27	1871
Superior parietal lobe	L	-22	-64	52	4.69	1705
Precentral gyrus	L	-46	–2	56	4.93	681
Superior medial gyrus	L	-4	64	20	5.36	412
Dorsolateral prefrontal cortex	L	-38	58	16	5.51	383
**Control condition**						
Precuneus	L	-16	-62	32	4.68	787
Supplementary motor area	L	-8	10	58	5.23	474
Inferior frontal gyrus	L	-42	20	8	6.04	344
*Anterior insula*	L	-30	18	12	4.49	

*Coordinates (mm) are in MNI space. L, left hemisphere; R, right hemisphere. Cluster-level, *p* < 0.05, family wise error corrected, voxel-level, *p* < 0.001, uncorrected.*

## Discussion

The present study used a modified version of UG to explore how social support modulates responders’ fairness-related decision-making processes and the underlying neural mechanisms. Behavioral results showed increased unfairness feelings and rejection rates for unfair offers in the social support condition compared to the control condition, suggesting that social support indeed impacted participants’ fairness considerations and responses. These results helped to identify which is a more reasonable explanation between two possible hypotheses: the “negative emotion buffer” hypothesis that social support might buffer participants’ negative emotional feelings elicited by unfairness and result in decreased rejection rates, or the “norm violation confirmation” hypothesis that social support might enhance participants’ awareness of fairness norm violations hence resulting in increased rejection rates. The current results were in line with the “norm violation confirmation” hypothesis. Participants reported higher level of unfairness feelings when getting social support from others, indicating that they became more sensitive to fairness norm violations, the motivation for rejection was enhanced. A recent UG study has found that self-affirmation can augment the responders’ psychological resources and increase their rejection rates of unfair offers (Gu et al., 2016). Given the emerging evidence that people can gain enough psychological resources from social support to cope with problems (Wilcox, 1981; Cohen and Wills, 1985; Delongis et al., 1988), our findings demonstrated that others’ support can act as powerful psychological resources and lead people a stronger a tendency to reject unfairness.

In fact, neither the behavioral results nor the neural results supported the “negative emotion buffer” hypothesis. This hypothesis would expect decreased activation in amygdala toward unfair offers being observed under the social support condition. However, decreased amygdala activation wasn’t observed in the interaction between context and unfairness or the interaction between context and response. It was increased amygdala activation (although at an uncorrected threshold) that was found during rejecting unfair offers in the social support condition compared to the control condition. The overall neural results were consistent with the “norm violation confirmation” hypothesis. Stronger neural activations in right AI and left dACC in the social support condition compared to the control condition. Further parametric analysis revealed increased activations in left AI and right dACC with unfairness feelings under the social support condition. The activation of AI observed in UG studies have been considered to be associated with the detection of norm violations, supported by the evidence of its involvement in signaling deviations from people’s expectations (Spitzer et al., 2007; Xiang et al., 2013; Zheng et al., 2015; Cheng et al., 2017; Vavra et al., 2017; Zinchenko and Arsalidou, 2017). The dACC was also suggested to be involved in detecting conflicts related to social expectation violations (Chang and Sanfey, 2013; Guo et al., 2014; Zheng et al., 2015; Vavra et al., 2017; Zinchenko and Arsalidou, 2017). The present behavioral results showed that unfair offers evoked participants’ higher unfairness feelings, indicating their stronger perception of fairness norm violations. Taken together, these data suggested that the responders experienced higher level of unfairness and detected stronger norm violations in the social support condition, resulting in greater AI and ACC activations. The increased activation of pgACC was revealed during receiving unfair offers in the social support condition. Similar result has also been found in another study of our group which focused on the impact of social pressure (Zheng L. et al., 2017). The pgACC has been thought to be engaged in person perception and mentalizing (Amodio and Frith, 2006). Considering the similar activations of pgACC in these two studies, this region might not involve in the processing of specific social situation, but the common communicative intentions during general social situations, future work is needed to probe the exact function of pgACC during such social contexts.

Additionally, accompanied with the increased rejection rates of unfairness in the social support condition, significant activations of AI and mPFC were identified in the interaction between response and context. Both regions were more active during rejecting than accepting unfair offers in the social support condition compared to the control condition. Existing studies have proposed that the AI activation was linked to the rejection response to unfair offers (Sanfey et al., 2003; Tabibnia et al., 2008; Kirk et al., 2011). Our results also proved the critical role of AI in unfairness rejection, which might imply that the responders perceived a stronger norm violation signal when they made a rejection decision. This finding provided further evidence that social support let the responder be more concerned about fairness norm violations. Consistent with previous studies that mPFC was more activated during the rejection than acceptance of unfair offers, the activation of mPFC in the current study was considered to be engaged in monitoring individuals’ behavioral responses (Civai et al., 2012; Corradi-Dell’Acqua et al., 2013).

To sum up, our findings provided both behavioral and neural evidence for the modulation of the responders’ fairness-related decision-making processes by social support. Behaviorally, the responders reported higher level of unfairness feelings and rejection rates of unfair offers when they received social support from others, indicating that they were more sensitive to fairness norm violations. Neurally, with other’s social support, increased activations were found in AI and dACC during processing unfairness, further implicating these two regions as being responsible for the detection of norm violations. The stronger activations in AI and mPFC were observed when rejecting unfair offers in the social support condition. In summary, the present study demonstrated that the fairness-related decision-making processes are context-dependent and are modulated by social support.

## Ethics Statement

This study was carried out in accordance with the recommendations of Ethical Committee of East China Normal University. Written informed consent was acquired from all subjects before the experiment. This study was approved by the Ethics Committee on Human Experiments of East China Normal University.

## Author Contributions

XG and LZ designed the experiments. LZ and XC programmed the experimental scenario and performed the experiments. CW, LZ, and XC analyzed the data. LL, CW, LZ, and LC joined in the interpretation of data. CW, LL, LZ, and LC carried out the writing. All authors read and approved the final version of the manuscript for submission.

## Conflict of Interest Statement

The authors declare that the research was conducted in the absence of any commercial or financial relationships that could be construed as a potential conflict of interest.
